# Phosphorus Acid—Its Compounds, and Their Therapeutic Value in Nervous Diseases

**Published:** 1874-12

**Authors:** T. Curtis Smith

**Affiliations:** Middleport, Ohio


### PHOSPHORUS ACID—ITS COMPOUNDS, AND THEIR THERAPEUTIC VALUE IN NERVOUS DISEASES.

BY T. CURTIS SMITH, M. D., MIDDLEPORT, OHIO.

(*Read before the Meigs County, Ohio, Medical Society.*)

GENTLEMEN,—I have chosen this subject for our consideration to-day for two reasons, viz: 1st. It is an agent of no mean value as a theraputic and dietetic remedy. 2d. It is an agent which we, as a Society, have not discussed.

It has, it seems to me, been too much overlooked, for upon giving it consideration we find it is an element, found in our tissues, especially in the brain and nervous systems, and as a proximate principle in bone, as phosphate of lime—bone (Dalton's Physiology, third edition, p. 74) containing 550, dentine 643, enamel of teeth 885, cartilage 40, muscle 2.5, blood 0.3, gastric juice 0.4 parts in a thousand. The urine, in health, yields 12.50 part (*loc cit.* p. 352) of the phosphates of soda, potassa, magnesia and lime, showing that it is quite freely introduced into the system, and is either carried out unassimilated, or, having been assimilated, is the result of gradual and continual oxidation in the tissues of which it has formed an element. Bile contains of these phosphates 15.24, pancreatic juice 0.45, milk 6.0 parts in a thousand. If we turn from these and inquire into the composition of the foods we eat to sustain the body, we will find phosphorous, in one form or another, as an element in many of them—ranging in qnantity from a mere fraction to 50 parts in a thousand. We find it in our meats, milk, fish, oat-meal and the bran of our flour. This latter fact should teach us to use the whole grain of wheat, and not throw away the hull, as is usually done.

By a careful selection of foods we can very often supply the system with an abundance of phosphorous, often a great desideratum.

Dr. Franklin (Medical and Surgical Reports, vol. xxiv, p. 494) made numerous experiments upon the development of fungus growth in potable waters, standing waters, sewage, etc., and came to the following conclusions concerning phosphorous:

1st. Potable waters mixed with sewage, urine, albumen and certain other matters, or brought into contact with animal charcoal, subsequently develop fungoid growths and other organisms, when small quantities of sugar are dissolved in them and they are exposed to a Summer temperature.

2d. The germs of these organisms are present in the atmosphere, and every water contains them after momentary contact with the air. (For an excellent statement, see extract from Professor Panceri in the *London Lancet* for April, 1872, p. 218, in which a different view is set forth from that by Dr. Franklin.)

3d. The development of these germs cannot take place without the presence of phosphoric acid, or a phosphate, or phosphorous in some form of combination. Water, however much contaminated, if from phosphorous, does not produce them. A German philosopher has said, without phosphorus, no thought," which may be now changed to "without phosphorus, no life."

We thus have a glimpse of the importance of this element in the composing ingredients of animal structures, and from it may readily infer the necessity of maintaining a sufficiency of it in *our own* structures, in health and disease; and may know that serious disease of the osseous and nervous tissues may result from an insufficiency of it in them, in some one of its combinations.

Let us now look at the physiological effects of this agent when therapeutically applied, or when taken in health:

Phosphorus is stimulant and aphrodisiac (Waring), and in large doses is irritant and poisonous, death being sometimes produced by small doses. Dr. Ainstie relates (*Practitioner*) a case in which very unpleasant effects followed the use of small doses ($\frac{1}{30}$ gr. *ter die*).—It acts on the kidneys, and as a sudorific. We need scarcely mention the irritant effects of the fumes of phosphorus, or that caries of the maxilla is caused by too free exposure to them, as these are facts known to every one.

Wood states (*Therapeutics*, 1865), it is stimulant to the nervous centres, strongly excites the sexual appetite, promotes the secretions, especially those of the skin and kidneys, but, being diffusible, it operates promptly and for only a short period. Given in medicinal doses, it excites a feeling of warmth in the stomach, fullness of pulse, heat of the skin, invigorates the mental functions and muscular power. Given in larger doses, it causes gastric irritation and tenderness, vomiting and purging—death, resulting from inflammation, gangrene and perforation of the stomach. Upon the system at large, poisonous doses cause rapid pulse, great nervous excitement, headache, vertigo, delirium, muscular pains and cramps, convulsions, and insensibility before death. Here we catch the fact, connected with so many of our valuable remedies, that its too free or inappropriate use is dangerous; while it may be invaluable under discriminating application. Phosphorus, whether combined or uncombined, is absorbed into the system when taken into the alimentary tract. Oil of phosphorus, when administered to dogs or cats, has been detected in the liver, and it has (Herron, Huseman and Maune

—*Am. Jour. Med. Science*, April, 1867, p. 529) been found in the livers of herbivorous and carniverous animals, even when given in very small doses ; and after large doses, the liver yields a phosphorus odor by masceration with sulphuric acid. It has also been detected in the muscular system and cardiac tissue. The action of too large doses of phosphorus, according to many writers, tends to produce fatty degeneration of the liver and kidneys, and, sometimes, of the heart and lungs. This fact is, however, disputed by others—thus making it a question *subjudice*. Still it stands out as a warning to us against its too free or indiscriminate administration.

Phosphorus is most active in its effects on the brain and nervous system, both of which "are rich in phosphorus" (Wood). To these tissues it stands "in the same relation that iron does to the blood," from which we may readily infer that phosphorus is as strongly indicated in nervous debility as iron is in anemia. This, however, remains to be clinically proven.

Phosphorus is eliminated from the system chiefly, if not entirely, by the kidneys. (See interesting statement by Dr. Moffit before the British Association for the Advancement of Science, on the influence of atmospheric pressure in relation to excretion of the phosphates and phosphoric acid—*Compend. Med. Science*, January, 1870, p. 88). Pereira says (*Therapeutics*), "Phosphorus is thrown out of the system as phosphorus acid, and perhaps also as phosphoric acid and phosphuretted hydrogen." But there is no lack of proof that the earthy phosphates are also thrown off in solution in the urine, as the following statements of the results of extended experiment by L. H. Wood, of New Haven, shows, (*Amer. Jour. Medical Science*, April, 1870, p. 507, review by J. Minus Hays). These statements, given to prove other conclusions, also serve our purpose here. We will commence with his fifth proposition, as given by his reviewer :

5th. "The total *phosphoric acid* exerted per hour is largest during the day, rising highest after the principal meal." * * * [Confirmed by other experiments].

6th. "The *alkaline phosphates*, when an ordinary diet is taken, are greater by day than by night; on a fixed diet, the reverse is true."

7th. The *earthy phosphates,* on the other hand, are largest in amount during the day, both on ordinary and fixed diets.

8th. The *total phosphoric acid* is very greatly affected by the amount and kind of food taken.

9th. The *variations* in the amount of phosphoric acid are not sufficient to afford any indication of the previous mental condition.

10th. The *alkaline phosphates* are only slightly increased on increasing the amount of mental labor.

11th. The *earthy phosphates* are *diminished* under the same conditions, by an amount varying from 20 to 40 per cent.

12th. No *such increase of phosphoric acid* as would be required by the theory of disintegration of nervous tissue during action was observed in these experiments.

13th. "The *alkalinity* of the day urine is not due to the presence of alkaline phosphates in excess."

In the foregoing quotation we have a clear view of how phosphorus is, for the most part at least, eliminated from the system, and in what combinations. We have the further view of the time when, and condition under which, the amount secreted is greatest or least. We still further have a fact set forth that seems to controvert the opinions of some physiologists, viz: that the greatest amount of the phosphates is eliminated after active mental labor; whereas the reverse is true. If this be so, it is but another proof that the use of the brain increases, rather than diminishes, its strength and activity, the same as use of the muscles gives them tonicity and strength.

All the physiologists that I have been able to consult fail to mention the elimination of phosphorus by the feces, by perspiration, or respiration; but writers of authority do speak of its elimination by respiration in cases of poisoning by it, as demonstrated by the lumiosity of the expired air. From this fact, we may, with others, conclude—

1st. That it is readily absorbed and taken into the system when thrown into the stomach, whether with food or as a medicine. This is confirmed by all observers on the subject.

2d. That its chief source of exit is by way of the renal organs.

Its effects, physiologically, on the system have been but meagerly mentioned; but in a practical paper, as this is designed to be, we have no room for further comment only as it shall occur in connection with its therapeutical applications. We will begin this part of the paper by commencing with the use of phosphorus in diseases of the nervous system, as this seems to us most proper.

In nervous *asthenia*, resulting from work, mental or physical, from exhausting diseases of the nervous system, and in cases of general nervous debility or prostration, attended or not with general emaciation, phosphorus is a most valuable agent. Testimony in its favor in this respect is, doubtless, not wanting among the members of our own society. I have repeatedly observed the rapid restoration to health in this class of cases in my own practice, even after a general course of tonics and stimulants had failed to produce the desired effect. A well marked case of this type, in which the nervous asthenia was well marked, and resulted from serious disease of the nerve centres, came under my observation during the last spring. During a long continued illness, the patient, a stout, robust man of middle age, had no appetite for any food, and the stomach being irritable, he could force down but little without its being followed by emesis. His pulse was feeble, skin relaxed, and had profuse perspiration whenever he slept, and always awoke with a feeling of complete exhaustion. The bowels were moved sufficiently often, and the urine was not scant, and always, by its heavy deposit and its character, indicated rapid nerve waste. He was unable to sit up or help himself, and feelings of great exhaustion followed immediately by profuse perspiration came on after physical efforts. Complicating this state was the presence of a large pulmonary abscess, from which he was expectorating large quantities of purulent matter every day. There was also a tendency to periodicity, as manifested by a chill every day, or every alternate day, which generally occurred in the morning, followed by fever and sweating. These were broken up by the free use of quinia and arsenic. Tonics and stimulants in every shape failed to greatly ameliorate the difficulties attending this case. I had attempted to give him phosphorus in two different forms and at different times, but failed to get a sufficient quantity into his system to be of any noticeable avail. He had, however, gained a little in the last two weeks, but by no means satisfactorily. At this stage of his case Dr. Knight and Fisher, of this Society, were called in council. They, with myself, agreed that the indications strongly pointed to the use of phosphorus, with such nourishment as could be taken, with the use of sufficient oil to use up the bile as it was secreted by the liver, and which would also greatly assist in building up the nervous system. Accordingly he was placed on strict orders of perfect quietude and the avoidance of all mental excitement and given the following: ℞.—Acid phosphoric dil., ʒi. every four hours, to be taken in a half ounce of pure ol. olive (salid oil); also to have a drachm of saturated solution of the hypophosphite of lime every four hours, intermediate with the preceding dose. A liberal allowance of milk was also ordered. The quinia, which he had used largely prior to this date was gradually dropped off, but had to be again renewed later to meet periodic manifestations. From or soon after the above plan of treatment was adopted the patient began to rally, and with the exception of a few drawbacks, caused by interloping complications, as a circumscribed pleuritis, followed by a pretty free secretion of pleuritic effusion, (which was afterwards removed by the use of digitalis internally and tincture of iodine over chest), occasional severe coughs, also occasional severe mental strains, caused by business complications, he continued to do well and has finally made a tolerable recovery. His appetite became very craving, and his weight increased much more rapidly than his strength, and doubtless his rapid gain for the time had as much to do with the absorption of the pleritic fluid effused as did the digitalis and iodine.

One cannot appreciate the gravity of a case of this type, and of as great severity, without seeing and having to manage it.

Dr. W. H. Broadbent, of London, (*Practitioner*, April, 1873, p. 230, also *London Lancet*), relates several very interesting cases of "Nervous Break-down from Over-work," in which he found phosphorus an invaluable remedy, as he did also in other cases of nervous debility from whatever cause, and in cases of neuralgia. He considers its action as analogous to arsenic, but much more rapid in producing its effects on the system, and much more available in many cases.

Dr. David Mack (*Boston Medical & Surgical Journal*, Feb. 12, 1874) relates a very interesting case, which he was pleased to denominate "A case of cerebral exhaustion," of extreme severity, in which there seemed to be great loss of nerve influence, and debility. Phosphorus was one of his chief remedies in the case, which, though tedious, finally recovered. This case is well worth perusal by any one of us.

There is no lack of testimony in its favor, in cases of these characters, for the agent is highly commended by the names of Radcliffe, Ainstie, Broadbent, Gubler, Asburton Thompson, Hammond and a host of others. Besides the above fact, its value in nervous debility is now recognized by, and taught in all, the leading Medical Colleges throughout our land and across the Atlantic.

Dr. John C. Thorowgood (*Compendium Medical Science,* January, 1870, p. 188) "thinks that phosphorus has a restorative action over weak nerve tissue, such as iron exerts upon anemic blood," and that it acts best in this difficulty when given pure.

Dr. Routh, of London, effectually relieved a case of partial cerebral exhauston by the use of phosphatic food and 10 grains of allotropic phosphorus daily, (*Medical & Surgical Reporter,* vol. xxix, p. 81, 1873), also a case of nervous irritability with a solution of phosphorus; and looks upon it as a nerve tonic. He commends very highly the phosphide of zinc as "a very elegant preparation" from which all the effects of phosphorus may be obtained. He uses it in pills of one-tenth grain each.

Without multiplying authorities regarding its value in nervous debility, we will turn to its use in neuralgia. In this affection I have had so little experience in its use that I cannot speak of its great value from personal knowledge. In two cases in which I resorted to it, there was evident benefit from its administration, while in a third case I could obtain no benefit from its extended use. This latter was the most obstinate case of neuralgia (trigeminal) it has ever been my lot to meet. It occurred in the person of a female, broken down in health and with greatly impaired nutrition, caused by hard labor and frequent parturition.

Dr. J. Ashburton Thompson, in an article on phosphorus in neuralgia, records eighteen cases, arranged in three different groups, or classes, viz: "Acute Primary Attacks," "Acute Recurrent Attacks," and "Chronic Cases." Ages of first class, 26 to 46 years; second class, 30 to 60 years; third class, 24 to 40 years. These comprised cases of trigeminal, cervico-occipital, cervico-bræchial and one case of recurrent sciatica.—(*Practitioner,* July, 1873, given in *Medical & Surgical Reporter*, 1873, vol. xxix). All the cases in the first two classes were cured, and three, or one-half of those in the third class—one of them having been afflicted for sixteen years, with very little intermission from pain; two others, though consumptive, were relieved, while the remaining one was not benefitted by it. He found that a few doses in the acute cases gave marked relief, and their cure was prompt, while the chronic cases required more time and more persevering use of the drug; yet in all cases where at all relieved, evidences of benefit followed the first few doses. He gave large doses, and says (*loc. cit.*), "to prescribe less than the one-twentieth of a grain in the first place is to render its therapeutic action apparently variable and uncertain," and follows with a recommendation of one-twelfth of a grain as a commencing dose, every four hours. He prefers a tincture made with absolute alcohol.

In 1863 (*British Medical Journal*, November, 1863), Dr. C. B Radcliffe published an account of four cases cured by the use of the hypophosphite of sodæ; two were cases of migraine, one trigeminal, and one sciatica.

Chapman only last year (work on "Neuralgia and Kindred Diseases," p. 267, 1873), says: "Phosphorus is, in my opinion, more likely to intensify than to palliate neuralgia."

Dr. G. M. Bradley (*British Medical Journal*, October, 1872), relates a case cured with it after every other means tried had failed. Broadbent also used it with success in this affection.

In a later article by Dr. J. A. Thompson, in the *Practitioner* for October, 1873, he relates, under the same divisions above mentioned, nine cases of "primary acute" attacks of neuralgia, all of which were promptly cured with phosphorus or its compounds; and eight cases of "recurrent" neuralgia, all of which were cured except one, which was relieved, and one case of this group where it returned (tragеminal) after three months. He also adds six more cases to his previous list of "chronic" cases. Of these, two were relieved and four recovered. He says: "In (loc cit) the course of the experiments of which the result as regards neuralgia alone is here published, I have observed cases of other diseases in which I believe further experience will show phosphorus, either pure or in one of the forms described above, to be a remedy most valuable and of somewhat wide applicability."

Dr. E. Slade-King speaks very highly of it in neuralgia, and in uncomplicated cases he "has found it most successful in its results, and has not noticed any unpleasant effects. Its action for good has been more rapid than any drug which was not narcotic or anodyne, and its results lasting in most cases."

Upon thorough investigation, I think it will probably be found that those persons who have failed to receive benefit from phosphorus in neuralgia, as a rule, have not taken enough of it. Formerly, one-twentieth of a grain of phosphorus was considered a large dose; now, the dose by good authorities is placed at from one-twentieth to one-fourth of a grain. I have myself given it with benefit in one-fourth grain doses after each meal, made into pill with pulv. glycerizæ, and have seldom observed any ill effects from its use. The same may be said of the phosphide of zinc. One of our able authorities directed its use in one-seventieth grain doses. This is simply nonsense. It is now commended in doses of one-twentieth to one-fourth of a grain. I now seldom order less than one-eighth grain, and find it valuable in such doses, but have found it to irritate the stomach sometimes in doses of one-fourth grain.

The combination of phosphate of lime is one of the most valuable we have, and one of the most reliable; and when a reliable syrup of the "lacto-phosphate of lime" can be had, it is pleasant as well as valuable. The various salts of phosphorus are all more or less valuable, and some of them are especially applicable to certain cases. Phosphorus is best administered in pill or in suet inclosed in gelatine capsules. Any tincture that can be made of it is nauseous in the extreme, and it is exceedingly difficult to get patients to take it. Even the pet formula of Thompson is too disagreeable for common use. A good formula for pills is—℞. Ext. glycerizæ, ℈ ii.; phosphorus, grs. i. M. ft. pil. x. to xx. (as may be desired.) S. One after each meal. Any vegetable extract may be substituted for the liquorice.

The phosphide of zinc is best given in pills with an extract or combined with other remedies not incompatible; but when the dose is placed below one-tenth of a grain, little effect need be expected from it.

Phosphorus pills in any form are liable to rapid deterioration on account of its rapid oxidation. The phosphide of zinc is not nearly so open to this objection.

Of the tinctures of phosphorus the following is one of the most pleasant and stable: (*Southern Medical Record*, 1873, *p*. 436.) ℞. Phosphorus grs. i.; chloriform ʒvi.; glycerine ℥ii. Mix. Sig. One or two drachms after meals. Here the chloroform is a great objection unless allowed to evaporate.

I deem some of the phosphates as by far the most easy and pleasant of administration, and of these I prefer the phosphate of lime or phosphide of zinc; and prefer for delicate stomachs the syrup lacto-phosphate of lime.

Dilute phosphoric acid is pleasant, but in my experience, is of doubtful value aside from its mere acidity. I think, with Broadbent, that there is no more sense in giving phosphoric acid, for the sake of its phosphorus, than there is in giving sulphuric acid, for the sulphur it contains. I have given phosphoric acid in one and two drachm doses with no more effect than would be derived from its acidity. This, I know, is directly contrary to the statements of most authorities.

I shall now rest your minds from further listening to this paper after giving the indications and contra-indications for the use of phosphorus, and naming its antidotes. It is contra-indicated in nervous affections during an hyperæsthenic state of the nerves or nerve centres, during an inflammatory state of the meninges, in exalted nervous irritability not connected with nervous æsthenia, in convulsions, cloric or tonic, in the phegmasiæ of all forms, in fevers and acute rashes of every kind.

"The indications for its use are the existence of disease (M. Gubler) unaccompanied by inflammation, fever, and (high) nervous excitation, and especially in such cases as are characterized by depression of the circulation either local or general, dimminished power of generating heat, exhaustion or local asthenia, with paralysis of sensation and movement."

Its antidotes are turpentine, carbonate of magnesia, charcoal, and mucilaginious agents. Turpentine is most frequently mentioned and used in counteracting its poisonous influence.

There are other wide fields for its application not here mentioned, as in paralysis, some forms of skin diseases, and nervous diseases of children, osseous diseasers, etc., but especially in pulmonary phthisis. But not wishing to tax your patience, I leave these points for future investigations and discussion. I therefore respectfully submit the foregoing for your consideration, and thank you for your kind attention.

